# Supplementary material for: Questionnaire and Portable Sleep Test Screening of Sleep Disordered Breathing in Acute Stroke and TIA
Source: J Clin Med. 2021 Aug 13;10(16):3568. doi: 10.3390/jcm10163568 (PMC8396899; doi:10.3390/jcm10163568)
Supplement: Supplementary file 1 [file jcm-10-03568-s001.zip › jcm-1239022-supplementary.pdf]

Supplementary Material S1. Survey 1: Berlin Questionnaire

Berlin Questionnaire (for sleep apnea)

Scoring Berlin questionnaire

Adapted from: Table 2 from Netzer, et al., 1999. (Netzer NC, Stoohs RA, Netzer CM, Clark K, Strohl KP.
Using the Berlin Questionnaire to identify patients at risk for the sleep apnea syndrome.
Ann Intern Med. 1999 Oct 5;131(7):485-91).

The questionnaire consists of 3 categories related to the risk of having sleep apnea.

Patients can be classified into High Risk or Low Risk based on their responses to the individual items and their overall scores in the symptom categories.

Categories and scoring:
Category 1: items 1, 2, 3, 4, 5.
Item 1: if '**Yes**', assign **1 point**
Item 2: if '**c**' or '**d**' is the response, assign **1 point**
Item 3: if '**a**' or '**b**' is the response, assign **1 point**
Item 4: if '**a**' is the response, assign **1 point**
Item 5: if '**a**' or '**b**' is the response, assign **2 points**

Add points. Category 1 is positive if the total score is 2 or more points
Category 2: items 6, 7, 8 (item 9 should be noted separately).
Item 6: if '**a**' or '**b**' is the response, assign **1 point**
Item 7: if '**a**' or '**b**' is the response, assign **1 point**
Item 8: if '**a**' is the response, assign **1 point**

Add points. Category 2 is positive if the total score is 2 or more points
Category 3 is positive if the answer to item 10 is 'Yes' OR if the BMI of the patient is greater than 30kg/m2.
(BMI must be calculated. BMI is defined as weight (kg) divided by height (m) squared, i.e., kg/m2).

High Risk: if there are 2 or more Categories where the score is positive
Low Risk: if there is only 1 or no Categories where the score is positive

Additional question: item 9 should be noted separately.

BERLIN QUESTIONNAIRE

Height (m) ________ Weight (kg)________ Age______ Male / Female
Please choose the correct response to each question.

CATEGORY 1

1. **Do you snore?**

_ a. Yes
_ b. No
_ c. Don't know

If you snore:

2. **Your snoring is:**

_ a. Slightly louder than breathing
_ b. As loud as talking
_ c. Louder than talking
_ d. Very loud – can be heard in adjacent rooms

3. **How often do you snore**

_ a. Nearly every day
_ b. 3-4 times a week
_ c. 1-2 times a week
_ d. 1-2 times a month
_ e. Never or nearly never

4. **Has your snoring ever bothered other people?**

_ a. Yes
_ b. No
_ c. Don't Know

5. **Has anyone noticed that you quit breathing during your sleep?**

_ a. Nearly every day
_ b. 3-4 times a week
_ c. 1-2 times a week
_ d. 1-2 times a month
_ e. Never or nearly never

CATEGORY 2

6. **How often do you feel tired or fatigued after your sleep?**

_ a. Nearly every day
_ b. 3-4 times a week
_ c. 1-2 times a week
_ d. 1-2 times a month
_ e. Never or nearly never

7. **During your waking time, do you feel tired, fatigued or not up to par?**

_ a. Nearly every day
_ b. 3-4 times a week
_ c. 1-2 times a week
_ d. 1-2 times a month
_ e. Never or nearly never

8. **Have you ever nodded off or fallen asleep while driving a vehicle?**

_ a. Yes
_ b. No

If yes:

9. **How often does this occur?**

_ a. Nearly every day
_ b. 3-4 times a week
_ c. 1-2 times a week
_ d. 1-2 times a month
_ e. Never or nearly never

CATEGORY 3

10. **Do you have high blood pressure?**

_ Yes
_ No
_ Don't know

Supplementary Material S2. Survey 2: Epworth Sleepiness Scale Questionnaire

Epworth Sleepiness Scale

Unique ID: ________

How likely are you to doze off or fall asleep in the situations described below, in contrast to feeling just tired? This refers to your usual way of life in recent times.

Even if you haven't done some of these things recently try to work out how they would have affected you.

Use the following scale to choose the most appropriate number for each situation:

0 = would never doze
1 = Slight chance of dozing
2 = Moderate chance of dozing
3 = High chance of dozing

Situation Chance of dozing

Sitting and reading ______
Watching TV ______
Sitting, inactive in public place (e.g. a theatre or a meeting) ______
As a passenger in a car for an hour without a break
______
Lying down to rest in afternoon when circumstances permit ______
Sitting and talking to someone ______
Sitting quietly after a lunch without alcohol ______
In a car, while stopped for a few minutes in the traffic ______

Total ______

Supplementary Material S3. Survey 3A: STOP-BANG Questionnaire

STOP-BANG Sleep Apnea Questionnaire

Chung F et al Anesthesiology 2008 and BJA 2012

**STOP**		
Do you **S**NORE loudly (louder than talking or loud enough to be heard through closed doors)?	Yes	No
Do you often feel **T**IRED, fatigued, or sleepy during daytime?	Yes	No
Has anyone **O**BSERVED you stop breathing during your sleep?	Yes	No
Do you have or are you being treated for high blood **P**RESSURE?	Yes	No

**BANG**		
**B**MI more than 35kg/m2?	Yes	No
**A**GE over 50 years old?	Yes	No
**N**ECK circumference > 16 inches (40cm)?	Yes	No
**G**ENDER: Male?	Yes	No

**TOTAL SCORE**		

Supplementary Material S4. Survey 3B: STOP Questionnaire

Appendix 1: STOP Questionnaire

Height _____ inches/cm Weight _____ lb/kg

Age _____ Male/Female BMI _____

Collar size of shirt: S, M, L, XL, or _____ inches/cm

Neck circumference* _____ cm

1. *S* noring

   Do you *s* nore loudly (louder than talking or loud enough to be heard through closed doors)?

   Yes No

2. *T* ired

   Do you often feel *t* ired, fatigued, or sleepy during daytime?

   Yes No

3. *O* bserved

   Has anyone *o* bserved you stop breathing during your sleep?

   Yes No

4. Blood *p* ressure

   Do you have or are you being treated for high blood *p* ressure?

   Yes No

* Neck circumference is measured by staff.

High risk of OSA: answering yes to two or more questions

Low risk of OSA: answering yes to less than two questions
